# Towards Sustainable Regenerated Cellulose Fibres: A Review of Conventional Spinning Technologies and Advancing Ionic Liquid–DMSO Solvent Systems

**DOI:** 10.3390/ma19204425

**Published:** 2026-10-21

**Authors:** Sophie Leah Fields, Ningtao Mao, Michael Edward Ries

**Affiliations:** 1 School of Design, University of Leeds, Leeds LS2 9JT, UK; sdsf@leeds.ac.uk (S.L.F.); n.mao@leeds.ac.uk (N.M.); 2 School of Physics and Astronomy, University of Leeds, Leeds LS2 9JT, UK

**Keywords:** cellulose, wet spinning, dry-jet wet spinning, ionic liquids, DMSO, regenerated cellulose fibres

## Abstract

Manufactured fibres account for over 70% of fibre consumption in the global textile market, with production centred on petrochemical-derived synthetics and regenerated cellulose fibres (RCFs). RCFs offer the opportunity to utilise alternative cellulose sources such as various plants, paper products, and postconsumer textiles, reducing the reliance on natural fibres such as cotton, which demands extensive land and water resources. The majority of RCFs are still produced using the viscose process, a method associated with high chemical, water, and energy usage. Employing ionic liquids (ILs) as an alternative solvent for cellulose dissolution is associated with desirable qualities such as the ability to capture and reuse the solvent, limited need for the pretreatment of cellulose, and alignment with green chemistry principles. At present, limited review articles have investigated the process of spinning fibres utilising ionic liquids as the dissolution solvent, particularly in combination with dimethyl sulfoxide (DMSO). To further understand the potential of RCFs, this review investigates existing cellulosic spinning technologies and the use of ILs, with particular attention given to IL–DMSO systems. By examining their combined behaviour and properties, this paper highlights their potential to advance sustainable textile manufacturing.

## 1. Introduction

The global production of textiles has grown by 3% annually over the past 40 years [1]. A substantial increase has occurred within the production of man-made fibres, which account for over two-thirds of all fibres. Most of these fibres are challenging to recycle and do not degrade at end-of-life [2]. Depending on the fibre source, each fibre has different properties that determine its performance and potential uses, which has led to increased innovation within textile research in response to sustainability concerns and limitations in production and technological advancement [3]. Based on existing forecasts, the demands for fibres will reach 240 million tonnes by 2050, of which 25 million tonnes will be harvested from cotton [4]. Cotton is the most prevalent natural and renewable fibre, yet it has a limit on its sustainability due to demand for crop land, pesticide use, and water consumption. An estimated 10% of the global water supply is used to grow cotton, evidencing the need to diversify the fibre market [4]. To overcome the restrictions and negative environmental challenges of synthetics and combine the benefits of natural renewable fibres, the development of RCFs have attracted attention in order to produce novel fibres with desirable environmental and performance properties [5].

Post-consumer textiles have significant impact on the environment. In total, 80–100 billion new items of clothing are manufactured each year, of which 80% are burnt or landfilled [6]. Of these disposed textiles, more than 90% are reusable, losing a vital feedstock that could be recycled into new materials through both mechanical and chemical methods [7]. Mechanical yarn-to-yarn recycling is calculated to cost 33.5% less than producing new cotton fibres, evidencing the economic benefits of fibre recycling in addition to reducing the demand for land to grow new cotton [8]. With chemical fibre-to-fibre recycling, used natural fibres can be dissolved and re-spun, to generate recycled fibres and yarns. It has been estimated that if 25% of cellulosic textiles could be recycled using chemical dissolution techniques, no further wood harvesting would be required for the spinning of viscose fibres [9]. However, as the production of viscose and lyocell fibres relies on wood-based feedstocks, as opposed to post-consumer textiles, there is a growing incentive to look for alternative RCF technologies.

Celluloses are polysaccharide biopolymers with a high molecular weight which can vary between different sources of either natural or regenerated cellulose. Native cellulose such as cotton and wood pulp generally exhibits high crystallinity, with tightly packed microfibrils arranged into the cellulose I allomorph, with a dense network that can restrict anion diffusion and lead to increased dissolution times [10]. In contrast, regenerated cellulose (cellulose II) contains more accessible hydroxyl groups and a disrupted lattice structure, enabling faster interactions with the solvents [11]. Celluloses are found in all natural plant fibres, together with hemicellulose and lignin that can influence both mechanical properties and dissolution behaviour [12]. These are often described as environmentally friendly due to their potential to be inexhaustible, endlessly renewable, and biodegradable at the end of life [13]. The polymer has the potential to be transformed into countless shapes and applications once extracted from the raw material source and is mainly used within the paper, packaging, and textile industries. At present, around 180 million tonnes of pulp are produced annually worldwide [14]. Cotton is the most prevalent natural cellulosic textile fibre [4] and is mostly composed of cellulose, accounting for 90% of the total fibre [15]. Due to its unique handling, lightweight, strength, and comfort properties, which are not matched by synthetic materials, cotton is found within 75% of all textiles, in addition to being a prominent material for making paper, medical textiles, and cosmetics [16].

An alternative fibre spinning system for RCFs is the use of ionic liquids as solvents for cellulose dissolution. These liquids possess desirable properties including high thermal stability, low vapor pressure, and efficient cellulose solubility [17]. ILs have been proven to demonstrate the ability to dissolve both virgin biomass and textile waste, evidencing a compelling route for chemical fibre-to-fibre recycling [18]. In addition to generating fibres made solely from cellulosic textiles, this technology has also been able to separate blended fabrics, as evidenced by Wu et al. facilitating the separation of cotton and polyester fibres [19]. However, at present, the use of ILs to spin RCFs has been demonstrated only at a laboratory scale, due to competition from more established spinning processes, high cost, and the rheological behaviour of the dope, which can limit stable extrusion, in comparison with established spinning processes [20]. To enable IL fibre spinning technology to become an industrialised system, solvent recovery, stable spinning solutions, and fibre consistency must be demonstrated, to show that the technology is environmentally and economically viable at scale [21].

## 2. Commercial Regenerated Cellulose Fibres

Regenerated cellulose fibres began to emerge in the fibre market during the 20th century, after the creation of artificial silk in 1884 by Duke Chardonnet, who discovered that wood could be used as a raw ingredient to make fibres [22]. At present, most regenerated cellulose fibres are made using viscose and lyocell processes using dry and wet spinning techniques. These processes convert native cellulose I into cellulose II, after which the chemical modifications cannot be reversed. Cellulose I is packed in parallel chains, whereas cellulose II is formed in an anti-parallel arrangement [23]. Cellulose II does not exist naturally, and it is formed through mercerisation or dissolution and regeneration [24]. These fibres account for 6.4% of the total textile market and are often derived from birch, bamboo, and eucalyptus trees; however, this in turn contributes to deforestation, in addition to employing harmful solvents to convert the wood into fibres [14].

Viscose or viscose rayon fibres are produced from a reaction between cellulose, sodium hydroxide, and carbon disulphide. Wood pulp is first mixed with sodium hydroxide (NaOH) and treated with carbon disulphide to produce cellulose xanthate. The cellulose xanthate is then dissolved in sodium hydroxide to produce a dope suitable for spinning viscose fibres [25]. However, more than two tonnes of highly corrosive chemicals are required per tonne of fibres produced, contaminated water and toxic fumes are released into the environment [26], and the process produces toxic by-products including hydrogen sulphide, which poses ecological and health risks and is challenging to recycle [25]. It is estimated that 60% of all greenhouse gas emissions produced in viscose processes result from the use and combustion of fossil fuels, whilst the remaining 40% comes from human activities, primarily deforestation [27]. At present, around 30% of the trees logged for the production of viscose come from endangered or ancient forests, and up to 70% of the total mass of wood is lost during the pulping process, evidencing that this current process has numerous negative consequences and operates on a linear, not circular, system [28].

The viscose process uses wet spinning techniques, in which the dope is extruded directed into the bath, allowing the polymer to coagulate immediately and solidify into a fibre [29]. The spinnability of the dope is specified as the ability to withstand the applied forces to adjust the fibre diameter without breaking the filament during the drawing process [30]. Key parameters for wet spinning include polymer concentration, the solvent(s)/anti-solvents, processing temperatures, solution flow rate and draw ratio, needle diameter, and winding speed [29]. Despite improvements to the handling of chemicals used to generate viscose fibres, the process still carriers environmental and health risks, 25–35% of carbon disulphide is not recoverable, and 200–300 tonnes of wastewater are produced for each tonne of fibre spun [31]. Additionally, more than two tonnes of chemicals are required per tonne of fibres produced, whilst contaminated water and toxic fumes are simultaneously released into the atmosphere [26].

Modal fibres are produced using a similar approach to the viscose process, using high quality beech or birch wood as the cellulosic feedstock rather than a blend of wood pulp [32]. Compared with viscose fibres, modal fibres offer increased dry and wet tenacities, higher wet modulus, lower water retention, and a decrease in the level of swelling. They are often referred to as ‘high wet modulus rayon’ and are primarily supplied by Lenzing [33]. An increase in carbon disulphide is included in the spinning dope compared with the production of viscose, in addition to modifiers and coagulation bath conditions. Once spun, the fibres are also drawn further and thermally fixed, giving the fibres their increased strength [34]. In addition, modal fibres have lower water retention capacity, and a greater resistance to alkaline exposure than viscose fibres [35]. Currently, the production of modal is more expensive than cotton or viscose, and the demand is expected to increase by 5–10% by 2030, due to the fibres’ popularity in the production of underwear and sportswear [35].

Lyocell is spun using a dry-jet wet spinning technique in which the solution is stretched through an air gap before coagulation’ Pulp from eucalyptus trees is used as the raw cellulose input [36]. Eucalyptus is used in the process due to having high levels of cellulose with relatively low lignin and hemicellulose, making it easier to convert into a suitable pulp [37]. The process was first developed in the 1960s but was not commercialised until the early 1990s [26]. The elongation of the dope in dry-jet wet spinning allows for greater molecular alignment, mechanical properties, and thinner fibres, due to the stretching of the dope travelling vertically through the air, and some of the solvents evaporate prior to immersion in the coagulating solvent [38]. This generally leads to increased tensile strength due to greater orientation of the cellulose chains [39]. The concentration of cellulose in *N*-Methylmorpholine *N*-oxide (NMMO) is generally between 8–12% (without the need for the pretreatment of cellulose) with 75–80% NMMO; however, by treating the cellulose with NMMO and water before drying and dissolution, an increased solution with a high concentration of 23% can be achieved [40]. Water, usually between 4–17%, is used in combination with NMMO for dissolving cellulose; this is incorporated in order to reduce the decomposition point of NMMO whilst allowing hydrogen bonding to occur between the NMMO and cellulose [22].

In addition to spinning lyocell fibres, dissolution technology using NMMO has led to the development of other cellulose products including films for packaging and medical materials [22]. More than 99% of the NMMO can be recovered and reused after spinning, allowing the re-use of the solvent in a closed-loop process [41]. NMMO works by disrupting the crystalline structure of cellulose. When mixed in an aqueous solution at 50 °C or above, the pulp crystallinity drops from around 60% to 20%. This pre-swelling of the cellulose suggests that it can facilitate a greater intensification of dissolution and higher molecular weight solutions can be designed and spun [42]. Upon regeneration, the crystallinity of lyocell fibres is about 60%; most regenerated fibres are found to be between 32–53% crystalline, with viscose around 40% [43].

Lyocell fibres often exhibit the most rounded and regular cross-sections of all fibres. This may be due to having a higher crystallinity than viscose fibres and a more consistent fibrillar structure from the skin of the fibre to the core [44]. The lyocell fibre spun by Lenzing, Austria is the most commercially available lyocell fibre with a fineness of 1.36 dtex, tensile strength of 672 MPa, and elongation of 19.6% [45]. The long crystals and highly orientated amorphous region lead to fibres having high dry and wet tensile strength [22]. Nevertheless, despite having increased mechanical strength, resistance to abrasion, and desirable handle properties, when exposed to a suitable environment, lyocell is able to degrade at a rate of 45–60% within 20 days [46].

The first research involving the recycling of cellulose-based textiles using NMMO was patented in 1997 by Firgo et al.; NMMO was utilised in the dissolution and regeneration of cotton waste textiles, which resulted in fibres with increased physical properties [47]. In 2017, REFIBRA™ technology, where waste cotton was incorporated with wood pulp, was introduced by Lenzing. This was the first RCF featuring recycled waste materials on a commercial scale, whereby up to 30% of the fibre could be made with waste cellulosic textiles, evidencing the potential to utilise post-consumer waste within chemical fibre-to-fibre recycling [48]. By reducing reliance on virgin cellulose feedstocks, such recycled regenerated fibres can also achieve improved lifecycle assessments and reduced overall environmental impacts [49].

Cuprammonium rayon or cupro fibres are spun through the dissolution of cotton linters or fibres into new materials and have long been considered an artificial alternative to silk [5]. The cellulose is dissolved in a solution of copper salts and ammonia before coagulating in a sulphuric acid bath. When first wet spun, the fibres are blue due to the copper and acid reaction; as the copper washes out of the fibre, the colour is lost [36]. Similar to viscose, the fibres are incredibly fine and have a silk-like appearance and touch, with a denier of around 1.33, compared to 2.5 for viscose [50]. However, despite the desirable aesthetics and fibre handle, cupro fibres have limited industrial application because the spinning process requires large volumes of chemicals and heavy metals, which can remain in the fibre and contribute to significant effluent pollution.

Although produced through a derivatisation route rather than direct dissolution, cellulose acetate fibres are a partially or fully acetylated derivative of cellulose. Formed of acetate ester, these fibres are produced by treating purified wood pulp with acetic acid, acetic anhydride, and a catalyst such as sulphuric acid [5]. Cellulose diacetate and triacetate are common forms of cellulose acetate with varying levels of crystallinity and biodegradation, with triacetate exhibiting the highest crystallinity as 92% of hydroxyls are converted to acetate groups, making the fibres highly hydrophobic [51]. Due to the thermal stability flexibility and transparency of cellulose acetate, the semi-synthetic polymer has many applications including textile fibres, cigarette filters, packaging, injection-moulded components, and photographic films [52]. To spin acetate fibres, the cellulose acetate is dissolved in a volatile organic solvent, usually acetone, before extruding directly into air, known as dry spinning, without the need for a coagulation bath, as the acetone rapidly evaporates leading to the solidification of the dope into fibres [43]. The production of cellulose acetate fibres is highly chemically intensive and the high acetylation properties can limit the fibres’ biodegradability, thus highlighting the economic and sustainability challenges associated with acetate fibres [52]. Yet, the spinning of regenerated cellulose fibres through derivatisation demonstrates the overall potential of cellulosic technology and the continued drive towards more environmental solvent systems.

For over a century, alternative solvents for the dissolution of cellulose have been explored for further development of spinning approaches. Recent advancements have focused on deep eutectic solvents, aqueous sodium hydroxide systems, and ionic liquids, each offering distinct advantages and limitations. Deep eutectic solvents have gained attention for fibre spinning due to low cost and biodegradability, but the cellulose dissolution capacity is temperature- and concentration-dependent [53]. NaOH systems such as NaOH–urea dissolve cellulose rapidly at low temperatures but require fast processing to avoid gelation, limiting their operational window [54]. In contrast, ionic liquids have emerged as versatile alternatives for fibre spinning due to their tuneable cation–anion combinations, hydrogen-bond disruption capabilities, and ability to dissolve cellulose without severe depolymerisation [11]. A wide range of ILs have been investigated in the production of fibres via both wet and dry-jet wet spinning techniques, which have demonstrated mechanical properties greater than those of existing fibres. Nevertheless, in order to be considered as a green alternative to the viscose process, high solvent-recovery rates of above 95% are required according to lifecycle analyses of IL-based cellulose spinning processes [55]. Despite promising fibre properties, ionic-liquid-derived fibres remain at pilot scale. There is presently no large-scale commercial equivalent to the lyocell process, creating uncertainty regarding operating costs and solvent stability under long-term continuous processing. Table 1 compares the properties and process methods of existing regenerated cellulose fibres.

## 3. Fibres from Ionic Liquids

Ioncell fibres, developed at Aalto University, are one of the key novel nextgeneration fibres spun using a superbase-derived IL; 1,5-diazabicyclo[4.3.0] non-5-enium acetate (DBNHOAc) is utilised as the solvent for cellulose dissolution [14]. Unlike traditional regenerated cellulose routes such as viscose or cuprammonium rayon, the Ioncell process is non-derivatising, low-emission, and closed-loop, requiring only water in the wash bath and avoiding the use of sulphuric acid or carbon disulphide [56]. Within the Ioncell process, cellulose is dissolved in 100% IL and extruded through an air gap before entering a water coagulation bath and drawn to form continuous filament fibres. Cellulose is often dissolved in ILs at elevated temperatures, as the decrease in viscosity favours mass transfer, thus enabling efficient dissolution [57]. In the Ioncell process, dissolution often occurs at temperature between 70–90 °C, with the spinneret between 70–80 °C, to enable the optimum required viscosity for the dope [58].

DBNHOAc is an SIL and has been confirmed to be a suitable solvent for cellulose dissolution, with the ability to dissolve wood pulp, paper, and cotton obtained from post-consumer textiles [59]. One of its most significant advantages is the liquid’s low viscosity, which makes the IL a suitable solvent for fibre spinning [60]. This rheological characteristic enables dissolution to occur at increased cellulose concentrations of 15–17 wt%, which exceeds the typical cellulose concentrations of 8–12% reported for many imidazolium-based ionic-liquid systems [21]. The ability to process higher cellulose contents can improve spinning productivity and reduce solvent consumption. A higher cellulose concentration, and thus, a greater number of molecular chains, facilitates increased crystalline connections, which often leads to an increase in the strength of the regenerated filament [61]. Furthermore, regenerated cellulose fibres produced from DBNHOAc solutions have been reported to exhibit mechanical properties that are competitive with, and in some cases superior to, those of commercially available regenerated cellulose fibres [14]. Ioncell fibres have reported to exhibit a tenacity of 58 cN/tex compared with 40–42 cN/tex for typical lyocell fibres [14,62].

Owing to the intrinsic amphiphilic nature of cellulose, the interaction and dissolution can occur differently with a range of solvents of varying polarity and hydrophobicity [63]. The cellulose for Ioncell is obtained from birch trees through a commercial hardwood prehydrolysis kraft pulp (PHK), which is dissolved directly into DBNHOAc [64]. PHK pulp offers low hemicellulose content and favourable molecular weight distribution, contributing to stable dope formation and strong regenerated fibres [65]. Compared with the lyocell process, which often uses a cellulose concentration of 10–15 wt% [66], the concentration of cellulose within an ionic liquid dope is typically between 8–12 wt%, depending on the IL used [67]. The high viscosity, strong chain entanglement, and sensitivity to water results in IL spinning dopes with a generally lower cellulose concentration than used in NMMO-based lyocell systems [68]. In addition, lyocell is produced on a commercial scale, with customised equipment [69], whereas studies into the spinning of IL fibres typically occur at a pilot lab-scale, representing a difference in processing environments and scale [70].

Acetate based ILs such as 1-Ethyl-3-methylimidazolium acetate (EMIMAc) have been proven to dissolve cellulose at high concentrations, whilst evidencing high solvent efficiency and lower toxicity, when compared to chloride based ILs such as 1-Butyl-3-methylimidazolium chloride (BMIMCl) [71]. Recent findings suggest EMIMAc possesses a higher number of ion pairs per unit volume than BMIMAc, contributing to its superior dissolution capability [72]. Acetate-based ILs have therefore attracted attention for their ability to dissolve cellulose, as the ILs can be captured and recycled after spinning RCFs. In addition, because the process can be carried out at low temperatures, a dry-jet wet-spinning method, similar to the lyocell process, was adopted to form fibres [25]. For dissolution within an IL, the optimum conditions of the cellulosic material must be of high purity, with minimal lignin and hemicellulose, low moisture, low to moderate DP, and minimal metals and extractives [68]. Optimum dissolution and spinning performance in IL systems generally requires cellulose with a DP of 300–800, which provides sufficient chain length for mechanical strength while avoiding the excessive viscosity associated with higher molar masses [13]. However, a variety of cellulose sources such as recycled newspapers and textile offcuts can be recycled using the Ioncell-F process; these can be sufficiently retained in the new textile fibres, depending on the dye type [73,74]. Following spinning, coagulation, and drying, Ioncell filaments are generally converted into staple yarns or blended with alternative fibre sources such as cotton, wool, or viscose [64].

Ioncell fibres, however, display exceptional mechanical properties, with elongation at break of 12% and tenacity of 50 cN/tex for the regenerated fibres of 15 wt% cellulose pulp [75]. At concentrations between 15–17 wt%, this led to the highest reported value for Young’s modulus at 34 GPa [13]. In addition, Ioncell fibres have demonstrated tensile strengths of 525–750 MPa in dry-state and 315–615 MPa in the wet state, ranking them among the strongest regenerated cellulose fibres reported [58]. The resulting IL-derived materials exhibit high strength, smooth surface morphology, and favourable dyeing behaviour, reflecting the uniform fibre structure, evidencing the potential of spinning RCFs from ionic liquids [58]. Although Ioncell fibres frequently exhibit higher tenacity and Young’s modulus than commercial lyocell fibres, it remains unclear whether the enhanced performance outweighs or offsets the higher solvent costs and process complexity. Consequently, future commercial processes may depend less on maximising fibre properties and more on demonstrating competitive solvent recovery rates, production economics, and process scalability. Table 2 shows further comparisons between different classes of RCFs.

## 4. Properties and Mechanisms of Dissolving Cellulose in ILs

Ionic liquids are often described as room temperature liquid salts; they are non-flammable, have low vapour pressure, good thermal stability, and are considered as a sustainable alternative to traditional solvents [60]. These liquids have the advantage of dissolving many polymers such as proteins including silk, keratin, chitosan, and polysaccharides without significantly reducing the material’s molecular weight [38]. A wide range of ILs with strong cellulose-dissolving capability have been developed, including imidazolium-based systems, quaternary ammonium ILs, quaternary phosphonium ILs, and superbase-derived ILs (SILs). SILs are formed by combining a superbase with a carboxylic acid which enables significantly lower production complexity and cost compared with conventional ILs [63]. The potential effectiveness of ILs as a solvent for the fabrication of films, fibres, and composites has been proven in recent studies, evidencing the potential for fabricating new cellulosic materials [38].

The physical and chemical properties of ILs can be adjusted by modifying the cation and anion structures [80]. It has been previously estimated that the number of potential cation and anion arrangements available equates to 10^12^ different ILs, evidencing a broad design space for tuning a variety of solvents [81]. The IL anion is often small and singly charged, with the anions’ activity highly dependent on charge, size, and polarity while dissolving [82]. One investigation by Villar et al. found that ILs with an acetate anion (OAc) were able to increase the rate of dissolution of lyocell fabric compared to alternative anions including diethyl phosphate (DEP), and dimethyl phosphate (DMP), demonstrating that the type of anion greatly influences dissolution and highlighting the importance of hydrogen-bond basicity [83]. In addition, ILs with anions such as acetate and phosphate possess lower viscosities that facilitate their use for various applications [84]. Ionic liquids containing imidazolium, pyridinium, ammonium, and phosphonium cations have been observed to dissolve cellulose when paired with a strongly basic, hydrogen bond-accepting anion, with current results imply that the higher the hydrogen bond basicity and dipolarity of the anion, the greater the dissolution of cellulose [81]. For cations, the viscosity increases with the increasing length of the alkyl chains partially due to stronger van der Waals interactions [85].

Tuneable cation–anion combinations and a strong ability to disrupt hydrogen bonding are features of halogen-based ILs such as 1-ethyl-3-methylimidazolium chloride (EMIMCl) and 1-butyl-3-methylimidazolium chloride (BMIMCl), which were some of the earliest ILs to demonstrate effective cellulose dissolution [86]. Chloride ILs demonstrate high viscosity and require elevated temperatures to reduce viscosity to workable levels; these conditions risk thermal degradation, discoloration, and reduced mechanical performance [87]. Acetate ILs including 1-Butyl-3-methylimidazolium acetate (BMIMAc) and 1-Ethyl-3-methylimidazolium acetate (EMIMAc) have been studied for cellulose solvation and fibre spinning and are the most common ILs used for dissolution [88]. These two ILs have the advantage of dissolving cellulose at a higher concentration, up to 16 wt% at 90 °C and 25%wt under microwave heating [84]. One investigation into temperature and pressure, by Moniruzzaman et al., concluded that the time required for complete dissolution of microcrystalline cellulose was over 16 h at 0.1 MPa, which was significantly reduced to 5 h at 2 MPa, whilst the temperature was maintained at 323 K [89]. While conventional heating enhances solubility, it also increases energy consumption; conversely, heating under pressure offers several advantages; it does not break covalent bonds, it is energy-efficient, it applies force uniformly, and it reduces dissolution times, making pressure an interesting factor to investigate for cellulose solubility [90].

Phosphate-based ILs exhibit excellent cellulose-dissolving capabilities and low viscosity; 1-Ethyl-3-methylimidazolium dimethyl phosphate (EMIMDMP), 1-Ethyl-3-methylimidazolium diethyl phosphate (EMIMDEP), 1,3-Dimethyl-imidazolium dimethyl phosphate (MMIMMP), and 1-Ethyl-3-methylimidazolium methyl-phosphate (EMIMMP), respectively, were prepared by reacting dimethyl phosphite with the corresponding imidazole in a molar ratio of 1.05:1 in a stepwise elevation to 130 °C for 10 h [17]. EMIMDEP was proven to demonstrate dissolution within a reduced time frame, with a 4 wt% cellulose solution completely dissolved at 11 min under 90 °C conditions [91]. Reduced viscosity facilitates improved mass transport, while the phosphate anion’s strong hydrogen-bond accepting character promotes the cleavage of intermolecular hydrogen bonding within cellulose, making phosphate-based ILs promising candidates for further exploration in cellulose dissolution and fibre-spinning applications [92].

The dissolution of cellulose in ionic liquids begins with the initial swelling, during which ions penetrate the fibre structure and disrupt established hydrogen bonds. Swelling occurs preferentially in amorphous regions, where the hydrogen-bonding network is less ordered and hydroxyl groups are more accessible [93]. Anions with high hydrogen-bond basicity, such as with acetate ILs, interact strongly with cellulose –OH groups, competing with and weakening the intermolecular hydrogen bonds that stabilise the cellulose microfibrils [81]. As swelling intensifies, microfibrils expand and the spacing between chains increases, facilitating the transition from surface-level disruption to full chain separation and migration into the solvent phase [11]. The rate and extent of swelling are therefore strongly influenced by cellulose crystallinity, hydroxyl accessibility, and the hydrogen-bond-accepting strength of the IL anion, with regenerated cellulose and highly basic anions exhibiting faster and more efficient swelling behaviour [84]. However, heating (often 80 °C to 100 °C) is typically required to lower the high viscosity of some ILs or IL mixtures to accelerate the diffusion of ions into the tight crystalline structure.

Dissolving cellulose in an IL relies on a synergistic cooperation between the anions and cations [31]. Small, localized anions (such as chloride or acetate) act as strong hydrogen-bond acceptors to form strong hydrogen bonds with some reactive groups of polymers e.g., hydroxyl groups of cellulose. These bind to the hydrogen atoms on the cellulose -OH groups, actively breaking the extensive internal network of hydrogen bonds that hold the crystalline polymer chains together and replacing the native inter- and intra-molecular hydrogen bonds of the cellulose chains [92,94]. Binding of the anions with cellulose also effectively gives the cellulose chains a negative local charge density, which keeps the individual cellulose polymer strands from re-associating [94].

The organic cations (such as imidazolium-based rings like BMIM^+^ or EMIM^+^) cluster around the newly formed anion-cellulose complexes to provide local electrical neutrality, and they help push individual polymer strands apart, preventing them from instantly re-aggregating [94,95,96]. The non-polar alkyl tails and aromatic rings of the cations interact with the glucose rings in the carbon backbone of cellulose via van der Waals and π–π interactions, helping to disrupt the sheet-like, solvophobic stacking of the carbohydrate rings. Bulky anions or excessively long alkyl chains on the cations weaken dissolution performance by slowing ion mobility and reducing localized charge density [26,92,94].

Anti-solvents act by interrupting the exchanges between cellulose and IL. The IL ions from hydroxyl groups collapse the swollen gel structure and promote re-aggregation of cellulose chains [97]. Water is particularly effective because of its strong hydrogen-bonding capability and high polarity; on the other hand, ILs are generally highly hygroscopic and can easily absorb water from the air [98]. Such an addition of water to an IL prior to dissolution can severely prevent cellulose solvation. Olsson et al. found that water concentrations 10 wt% in 1-ethyl-3-methyl-imidazolium acetate ‘largely inhibited dissolution’ [99]. Although ILs do not possess a true aqueous pH, the effective acidity or basicity is governed by water content, in addition to anion strength and cation structure [100]. Small amounts of water within the IL can alter the solvating power by competing for hydroxyl sites and altering ion mobility, which in turn disrupts dissolution behaviour and regeneration [101]. Although the hydrogen-bond disruption mechanism is the most widely accepted explanation for cellulose dissolution in ionic liquids, recent studies suggest that hydrophobic interactions, amphiphilic effects, and solvent structuring may also contribute. Therefore, a complete molecular understanding of cellulose dissolution remains an active area of research.

## 5. Dissolution of Cellulose in ILs with DMSO as a Cosolvent

Combining ILs with polar aprotic co-solvents including dimethyl sulfoxide (DMSO), dimethylformamide (DMF), and dimethylacetamide (DMAc) has been studied to observe cellulose dissolution. Compared with the use of neat ILs, the incorporation of co-solvents can enhance dissolution rates, improve heat and mass transfer, and facilitate processing at lower temperatures [55]. Among these cosolvents, DMSO been identified as the most suitable co-solvent, due to possessing highly polarity and an aprotic nature, which enhances the solvation power without disrupting or preventing the interactions between the IL and cellulose chains [102]. Furthermore, DMSO is a by-product from paper production, thus contributing to the sustainability and of the dissolution system whilst simultaneously reducing the costs involved in production [103].

The addition of DMSO can significantly reduce viscosity and enhance solvent penetration into the cellulose polymers. By introducing the DMSO to the cellulose first, before introducing the IL, the DMSO can pre-swell the cellulose to facilitate increased diffusion and accelerated dissolution, increasing solvent diffusion into amorphous regions of the cellulose and facilitating disruption of intermolecular hydrogen bonding [104]. A study of the rheology of cellulose solutions in ionic liquids EMIMAc, EMIMCl, and BMIMAc containing a high DMSO fraction of 75% evidenced a fifty-fold decrease in viscosity compared to pure IL systems [105]. Consequently, dissolution is accelerated and higher cellulose concentrations can be achieved compared with IL-only systems.

When an IL–DMSO solvent system was applied to spin RCFs, the incorporation of DMSO into EMIMAc was shown to produce a more homogeneous spinning dope, improving fibre extrusion, morphology, and mechanical properties [57]. Owing to the reduction in viscosity resulting from the addition of DMSO, using ILs with DMSO for fibre spinning has demonstrated increased stability and response to elongational forces, allowing increased fibre control and shaping during fibre extrusion. [106]. In addition, upon coagulation and drying, fibres produced from the combination of DMSO with EMIMAc were reported to have a denser internal structure and smoother fibre skin surface [79]. This was due to slower diffusion of the IL in the solution fibre into the water coagulation bath because of the DMSO, thus leading to slightly higher crystallinity and reduced amorphous regions. Investigations into the combination of IL–DMSO were undertaken to further understand the viscoelastic properties, concluding that all the solutions showed a shear thinning behaviours at high shear rates [107,108,109].

The effects of combining DMSO with multiple ILs, including acetate- and chloride-based ILs, on the cellulose dissolution and fibre spinning were evaluated by Kosan et al. [92]. Fibres spun from chloride-based ionic liquids were shown to demonstrate higher tenacity and lower elongation values than fibres spun from acetate anion fibres; however, acetate anions showed increased dissolution efficiency at higher cellulose concentrations [92]. Similarly, Zhu et al. (2018) demonstrated that cellulose spun from 1-ethyl-3-methylimidazolium diethyl phosphate (EMIMDEP) with a DMSO system yielded fibres with a high orientation (0.84) and a Young’s modulus of 41 GPa, representing values among the highest reported for cellulose fibres produced via IL systems [78]. The exceptional mechanical properties were attributed to an increased cellulose concentration, (23.4 wt%), which enabled improved chain interactions, and a higher molecular alignment during the drawing process, which could not be achieved using solely ILs [78]. This can be directly compared to research conducted by Zhu et al., where similar fibres were prepared without DMSO [110]. Microcrystalline cellulose was dissolved in EMIMDEP, at concentrations between 12.4 and 18 wt% cellulose, with concentrations above 15 wt% showing strong anisotropic patterns and isotropic patterns evident below 12.4 wt% [110]. Fibres spun at a concentration of 18 wt% possessed a tensile strength of 305 MPa and a Young’s modulus of up to 22 GPa, almost half the values of the fibres spun in combination with DMSO [78,110].

Furthermore, IL–DMSO systems have demonstrated the ability to dissolve a wide range of cellulose feedstocks, including microcrystalline cellulose, industrial cotton waste, post-consumer textiles, and blended fabrics, across a variety of IL–DMSO ratios. Successful cellulose filaments were obtained from the dissolution of an industrial cotton offcuts in EMIMCl and EMIMAc, using DMSO as the cosolvent in the mass ratio of 2:1 [75]. Furthermore, blended fabrics were recycled by selectively dissolving the cotton from the waste poly-cotton fabric using AMIMCl/DMSO and 1-ethyl-3-methylimidazolium diethyl phosphate (EMIMDEP)/DMSO systems. The results showed that when the mass ratio of EMIMDEP/DMSO was 1:1, the cotton content was 1.4 wt%, the dissolution temperature was 80 °C, and the time was 60 min, the cellulose demonstrated effective dissolution. [19]. In addition, the alkyl chain length of the cation in the IL can influence the viscosity and capacity of the hydrogen bonding, which can greatly impact the dissolution. It was found that cellulose dissolution can be facilitated by a longer cation alkyl chain of the IL cation at high IL–DMSO molar ratios (1:10, 1:5, and 1:2) but it is impeded at a low DMSO/IL molar ratio (1:1) [55]. If the DMSO fraction becomes too high, undissolved or partially swollen cellulose can remain within the solution due to the reduction in concentration of IL ions available to interact with the cellulose hydroxyl groups [55].

The addition of DMSO into ILs for cellulose dissolution can therefore improve the total solubility, reduce viscosity, shorten dissolution times, and lower the required temperatures for the formation of RCFs. Despite these advantages, however, DMSO possesses greater volatility than ionic liquids and can be lost during dissolution and solvent recovery processes due to its relatively high vapour pressure [17]. This may increase solvent recovery requirements and associated industrial costs. Additionally, toxicity considerations and process optimisation remain important challenges for the large-scale implementation for IL–DMSO-based cellulose recycling and fibre production technologies [17].

## 6. Relationship Between Cellulose Dissolution, Rheology, and Fibre Spinning

Despite substantial progress in understanding cellulose dissolution and fibre spinning in ionic liquid systems, a significant knowledge gap remains regarding the quantitative relationship between solution rheology and final fibre performance. The rheological behaviour of the spinning dope forms a critical link between dissolution and filament properties, as it determines how dissolved cellulose chains respond to shear and extensional forces during spinning. During dry-jet wet spinning, the dope is subjected to shear deformation during extrusion and extensional deformation within the air gap. These forces promote molecular orientation and chain alignment prior to coagulation. In turn, these flow-induced stresses directly influence the resulting structure and morphology of the filament [64]. Subsequent solvent exchange in the coagulation bath determines the crystalline and amorphous regions within the filament [54]. The presence of an air gap in dry-jet wet spinning enables controlled stretching and partial orientation of the cellulose chains prior to coagulation. The stabilising of the dope in suspension before it enters the coagulation bath produces fibres with a smooth surface, uniform wall thickness, and reduced skin–core formation [111].

The behaviour of the spinning dope is often influenced by the high viscosity of ionic liquids. Pure ILs are Newtonian liquids with an already elevated viscosity, with values ranging from 100 to 500 mPa s at 25 °C [68]. Among these solvents, ionic liquids containing halide anions are recognised as the most viscous, often displaying viscosities that are two to three orders of magnitude greater than those of conventional commercial organic solvents [112]. Cellulose mixtures in ionic liquids frequently have an increased high viscosity, even at elevated temperatures, leading to high energy consumption for efficient dissolution [107]. While ionic liquids are capable of dissolving cellulose at relatively high concentrations, the resulting solutions often exhibit high viscosities and complex viscoelastic behaviour. In turn, this can limit stable extrusion, increase flow resistance, and decrease continuous filament formation [13]. When ILs and cellulose are combined (where concentrations of 10–20% and higher have been reported), the viscosity can in addition increase further; the optimum viscosity for a spinning solution is between 1–10 Pa·s [57]. Excessively low viscosities can cause filament breakage, whereas highly viscous solutions lead to unstable flow, increased energy consumption, and difficulties during extrusion, as a result of excessive chain interactions [68].

Although numerous studies have investigated the combination of ILs with cosolvents for dissolution, limited studies into viscoelastic solution behaviour have been reported with regard to how this directly influences fibre spinning [107]. One investigation by Tarasova et al., observed the rheology and dissolution capacity of cellulose in 7-methyl-1,5,7-triazabicyclo[4.4.0]dec-5-enium acetate (mTBNHOAc) with a range of cosolvents including γ-Valerolactone (GVL), dimethyl isosorbide (DMI), and *N*,*N*′-dimethylpropyleneurea (DMPU) [107]. In that study, the viscosity of all solutions decreased with increasing temperatures, as expected, and it was concluded that the flow activation energy of cellulose in the IL/co-solvent systems was lower than that in pure IL [107].

Understanding the flow behaviour of biopolymer solutions is essential, as it directly influences conditions and the ability to produce materials that demonstrate efficient processability and desirable material properties [113]. First drawing the solution within an air gap during dry-jet wet spinning introduces extensional deformation, as the reorientation of cellulose polymers is connected to the extrusion of the dope in suspension. The molecular alignment and orientation is established during elongational flow, which is key in determining effective filament stability [114]. As a result, dry-jet wet-spun regenerated cellulose fibres typically exhibit greater molecular orientation and superior mechanical properties compared with conventional wet-spun fibres [115]. Further physical process parameters also influence the final fibre. Michud et al. (2016) demonstrated that extrusion velocity, draw ratio and coagulation conditions directly influence the resulting fibrillar structure, orientation and tensile strength of regenerated cellulose fibres [64]. A draw ratio of six was utilised to spin fibres from cellulose dissolved in DBNHOAc, with recorded tenacities above 40 cN/tex and a tensile strength of 600 MPa. That study also employed a water coagulation bath set to 15 °C, which was found to slightly promote greater orientation of the fibres [64].

Furthermore, the choice of antisolvent can also have a pronounced impact on the structure and properties of regenerated cellulose. Water, ethanol, or acetone can all be utilised within the coagulation bath for regeneration. One report by Tan et al. (2019) demonstrated that fibres coagulated in water, following dissolution in EMIMAc, exhibited a greater degree of crystallinity (43%) compared to cellulose coagulated in ethanol (21%), signifying the strong hydrogen bonding capability of water [116]. An investigation into coagulation baths containing a combination of IL–water was reported by Guizani et al.; the material was found to show increased mechanical properties. At a draw ratio of 12, the highest tenacity was observed for 30 wt% IL DBNHOAc in the bath, while the lowest was observed for 15 wt% IL in the bath. Fibres regenerated in IL-containing baths showed less pronounced fibrillar structure than fibres coagulated in pure water [115]. As ILs are hydrophilic and water soluble, the solvent diffuses out from the fibre, into the water bath, whilst re-establishing the original hydrogen bonds between the cellulose chains [117].

It has also been established that the spinneret diameter has a substantial impact on the filament shape in the air gap. A wider diameter spinneret leads to slower cooling of the air gap, causing a diminution in stress upon the filament and intrinsic properties before entering the coagulation bath. An increase in the spinneret diameter of 50% caused a decrease of 48.6% in elongational viscosity, as reported by Michud et al. [64].

Stable spinning conditions for all spinning methodologies promote molecular orientation and the formation of organised crystalline and fibrillar structures, which in turn can improve properties including tenacity, tensile strength, and Young’s modulus [29]. Consequently, successful fibre spinning depends not only on cellulose dissolution, but also on the coagulation bath, spinneret, and ability of the spinning dope to respond to shear and extensional forces during drawing and coagulation. At present, however, there are limited reports in the literature on predicted fibre properties when using a specific cellulose solution with a given viscosity and extensional viscosity. Future research should focus on establishing relationships between rheology, molecular alignment, coagulation kinetics, and fibre performance, in order to enable the development of predictive models for RCF spinning.

## 7. Challenges of Using ILs to Spin Regenerated Cellulose Fibres

As a novel area of spinning cellulose fibres, several factors and challenges remain, which hinder this technology with regard to both scale-up of fibre production and creating a standardised set of methods and requirements for producing IL-based cellulose fibres. At present, the lyocell process is widely regarded as one of the most environmentally favourable routes for regenerated cellulose fibre production due to the efficient recovery and reuse of solvent within a closed-loop process [66]. In addition to the low chemical costs, NMMO costs approximately GBP 2–4/kg, whereas common cellulose-dissolving ILs such as imidazolium, phosphonium, or superbase systems range from GBP 80 to over GBP 300/kg depending on purity and scale [57]. In order to bring prices down and improve solvent efficiency, the recycling of ILs is currently being investigated through technologies such as distillation, extraction, and membrane filtering, thus allowing the solvents to be used multiple times [118]. At present, reviews of IL-based cellulose processes typically report 95% recovery in lab or pilot setups; this figure does not yet match lyocell’s 99%+ industrial figures, partly because there is no full-scale IL fibre spinning facility and the material is not available on a commercial scale [70].

The solubility of cellulose is dependent on the processing parameters, including the time for dissolution, dissolution temperature, degassing of the solution, and the degree of polymerisation of the cellulosic source. Despite research into these areas, fewer studies have systematically examined the coagulation and regeneration stages, and the roles of anti-solvents, despite their influence on fibre structure, crystallinity, mechanical performance, and recyclability [97]. Computational studies demonstrate that regeneration involves interactions between cellulose hydroxyl groups and the chosen anti-solvent molecules; H-bonding plays a paramount role in both dissolution and regeneration, strongly impacting the crystallinity and formation of cellulose II upon coagulation [119]. In addition, the diffusion rate of the chosen anti-solvent into the IL and cellulose solution determines the speed of phase separation. Rapid diffusion facilitates dense, uniform structures, whereas slower diffusion can lead to more porous morphologies [119]. Quantitative studies further confirm that water penetrates IL cellulose solutions with diffusion coefficients in the order of 10^−9^ m^2^/s, approximately one order of magnitude higher than ethanol and nearly two orders higher than 2-propanol [120]. Therefore, additional optimisation of the regeneration process and studies into the coagulation conditions are equally as important as the dissolution, as mass-transport and anti-solvent behaviour can directly result into variations in crystallinity, pore structure, and mechanical properties of the RCFs.

Due to their limited biodegradability and moderate to high aquatic toxicity, ILs must be disposed of under strict environmental conditions. Wastewater containing ILs must go through activated-carbon adsorption, ion-exchange purification, membrane separation, or advanced oxidation processes before release as effluent into the watercourse [121]. Water is the most common anti-solvent used to coagulate cellulose dissolved within an IL; however, the strong interactions between ILs and water result in a significantly higher demand for energy in order to separate the two liquids compared to the removal of ILs from other solvents [122]. In addition to being significantly cheaper than alternative antisolvents, water produces more crystalline structures, with 43% crystallinity in water, compared with 13.5% in ethanol and 21.3% obtained using a treatment with ethanol and water [116]. When observing the coagulation of cellulose in water from a range of solvents, it was found that the coagulated cellulose gels were transparent using BMIMCl, translucent using EMIMDEP, and opaque using NMMO·H_2_O; coagulation was conducted through immersion into 35 °C water for 1 min; these differences in appearance were due to different void structures formed in the regenerated cellulose [123].

However, there is also the potential of small impurities or cellulose molecules remaining within the recycled ILs, which could alter the chemical properties and dissolution potential of the solvents [124]. Zhou et al. (2020) reported that cellulose experienced 31.9% degradation after being heated in 1-allyl-3-methylimidazolium chloride (AmimCl) at 90 °C for 72 h, with over 2% glucose discovered in the recovered ionic liquid [125]. Research by Elsayed et al., (2020) included spinning fibres using ILs that had been recycled multiple times; a five-cycle trial found that dissolved 13 wt% cellulose in recycled mTBDHOAc was not impaired during the dissolution [126]. On the other hand, an investigation into the reuse of recycled DBNHOAc, including a single reuse of the solvent for Ioncell fibre spinning, found that only 76.5% cellulose dissolution was achievable compared to its initial dissolution capability, due to the degradation of the chemical structure of the liquid and residual water content [118].

Another class of solvents has also emerged with the capacity to dissolve cellulose are deep eutectic solvents. DESs have similar physicochemical qualities to ionic liquids, in addition to low volatility and strong hydrogen-bonding interactions, which can facilitate disruption of the extensive intermolecular hydrogen-bonding network within cellulose [53]. However, in order to produce sufficiently stable dope flows, the spinneret diameter needs to be at least 0.5–1 mm, compared to conventional wet spinning processes using spinnerets with diameters of 10–300 μm, which are prone to clogging due to the behaviour of the dope, which is generally of high viscosity [53]. The requirement for larger spinnerets restricts achievable draw ratios and limits the degree of molecular orientation that can be imparted to the cellulose chains during elongational flow [127]. Furthermore, recent reviews indicate that the cellulose dissolution capacity of most DES systems remains substantially lower than that achieved with leading ionic liquid solvents, limiting their immediate applicability for fibre-spinning processes [128].

In order to improve solvent circularity, it may be desirable that a single IL should be optimised for the production of cellulose fibres and additional regenerated materials such as films, nonwovens, and packaging, resulting in an established system for producing a range of cellulosic materials from a primary solvent [129]. In addition, the source of cellulose can impact the dissolution and spinning processes. If utilising post-consumer textiles as the cellulosic feedstock, the finishes and dyes that have been applied may impact and disrupt the dissolution and spinnability of new fibres, and these may need to be removed before dissolution [130]. Inefficient dye removal can lead to a decrease in the mechanical properties of the fibres and undesirable spinning conditions; however, Haule et al. were able to prove that fabrics stripped of easy-care finishes and dyes and spun into regenerated fibres exhibited mechanical and surface morphology comparable to lyocell [47]. Insoluble dyes have been found to impact the spinnability of cellulose, necessitating the addition of virgin fibres or wood pulp for a homogenous dope solution [49]. Future industrial implementation will depend on balancing solvent performance with economic and environmental sustainability rather than maximising dissolution capacity alone [73]. When using ILs to produce RCFs, these fibres can reduce the consumption of carbon disulphide compared with viscose fibres; however, the long-term environmental profile of IL-derived RCFs depends on the solvent recovery and recyclability, degradation behaviour, and wastewater management.

## 8. Conclusions

Cellulose offers a promising alternative for many applications, with potential to replace existing unsustainable practices. Acceleration towards renewability, reduced emissions, and product traceability sits alongside the United Nations Sustainable Development Goals to create products with lower impacts and greater efficiency [13]. The possibilities of green solvents and the variety of sources of cellulose materials means there is still research being undertaken for fine-tuning regenerated fibres with desirable properties. The potential of recycling post-consumer cellulosic textiles from natural-based materials into regenerated cellulose fibres offers a promising solution to reduce the volume of products sent to landfill. As highlighted in this review, a range of solvent systems can be applied to manufacture RCFs, with particular focus on IL–DMSO systems that combine the strong cellulose-dissolving capability of ionic liquids with the viscosity-reducing effect of DMSO, representing a particularly promising approach for the development of scalable and sustainable regenerated cellulose fibre production. The market for regenerated cellulose fibres is expected to grow due to increased awareness of the recyclability, renewability, and biodegradability of such cellulose-based materials [131]. One central obstacle to the adoption of industrial scale IL processing is the price of ILs compared to existing chemicals [118], leading to a lack of large-scale research, and these solvents are often made in small quantities. In addition, there are still gaps in the literature with regard to the number of times fibres can be recycled using this technology; the possibility of recycling fibres multiple times using the binary system of IL–DMSO also needs to be explored [18]. While ionic liquids are often considered green solvents due to minor vapour pressure and low volatility, this does not guarantee sustainability. The environmental burden associated with solvent synthesis, purification, and end-of-life treatment remains insufficiently measured for many ionic liquids.

For recycling or converting native cellulose into fibres, there are still further investigations to be made into the interactions between IL–DMSO systems. While combining the two solvents demonstrates significant potential through reduced solution viscosity, increased cellulose concentration, and improved spinnability, their long-term industrial viability remains uncertain. There are questions regarding the desired DP for fibre spinning, solvent recapture, the range of cellulose used to create the fibres, and the potential of multifilament and dyed fibres. The removal of ionic liquids from the fibres following coagulation and washing is crucial to fibre purity, crystallinity, mechanical performance, and the possibility of closed-loop IL recovery. Fibres spun using a variety of ILs have been described; however, the majority of cellulosic inputs are derived of wood pulp, evidencing that there is still potential for fibres to be spun using alternative sources of cellulose as the raw material. Most of the literature cited in this study focuses on single-filament fibres and laboratory-scale systems, but commercialising of these fibres requires multifilament and continuous spinning technology. Consequently, future development should focus not only on improving dissolution efficiency and fibre performance but also on economic assessments, life-cycle impacts, solvent recyclability, and process scalability to determine whether IL–DMSO systems can offer a genuinely sustainable alternative to established lyocell technology. In summation, further research is still required for the development and optimisation of spinning fibres using ionic liquids, to progress to a more sustainable and circular textiles economy.

## Figures and Tables

**Table 1:** Comparison of Regenerated Cellulose Fibre Types.

Fibre Type	Structure and Properties	Raw Materials	Spinning Process	Chemicals Used	Environmental Concerns	References
Viscose	Semi-crystalline, good dry strength, low wet strength	Dissolving Grade Wood Pulp	Wet Spinning	CS_2_, NaOH, H_2_SO_4_	High chemical usage, toxicity, deforestation, high wastewater	[25]
Modal	Highly crystalline, high wet tensile strength	Wood Pulp—Beech, Birch	Wet Spinning	CS_2_, NaOH	Improved from the viscose process but still high chemical usage	[35]
Lyocell	Highly crystalline, with high strength, both dry and wet	Wood Pulp—Eucalyptus, Bamboo	Dry-Jet Wet Spinning	NMMO	Closed-loop, low emissions, low water use	[22,38]
Cupro	Fine, silky, highly uniform	Cotton Linters	Wet Spinning	Cuprammonium hydroxide	High water use, toxicity concerns from copper effluent	[43]
Cellulose acetate	Thermoplastic, smooth handle	Wood pulp—Acetylated cellulose	Dry Spinning	Acetic acid, acetic anhydride	Low toxicity but solvent emissions require control	[52]
IL fibres	Tuneable crystallinity, high tensile strength depending on solvent systems	Wood Pulp, Cotton, Textile Waste	Dry-Jet Wet Spinning or Wet Spinning	Ionic liquids (EMIMAc, DBNHOAc), DMSO	High circularity potential, low toxicity, potential for solvent recovery	[13]

**Table 2:** Comparison of Regenerated Cellulose Fibre Types and Methods.

Fibre Type	Tenacity (cN/TEX)	Tensile Strength (MPa)	Young’s Modulus (GPa)	Elongation at Break (%)	Key Advantage	Key Limitations	References
Viscose	20–30	250–450	5–11	15–25	Established production routes and low fabrication costs	High chemical use and poor wet strength	[5,43,54]
Modal	35–45	450–650	5–10	10–20	High wet tensile strength than viscose	High chemical use and reliant on carbon disulphide	[33,76,77]
Lyocell	35–45	500–700	10–15	10–20	Closed-loop solvent recycling system, high tensile strength	Prone to fibrillation and reliance on wood pulp feedstocks	[45]
Ioncell	50–61	525–750	<34	<12	High tensile strength, solvents can be recycled	Limited production scale and costs of chemicals	[13,21,58,75]
IL and DMSO	35–55	500–800	<41	5–15	Reduced viscosity, leading to higher cellulose concentrations and orientation, reduced costs	Solvent recovery and separation post spinning from the coagulation baths	[75,78,79]

## Data Availability

No new data were created or analyzed in this study.

## Abbreviations

The following abbreviations are used in this manuscript:

RCF	Regenerated cellulose fibre
IL	Ionic liquid
DMSO	Dimethyl sulfoxide
NaOH	Sodium hydroxide
NMMO	*N*-Methylmorpholine *N*-oxide
SILs	Superbase-derived ILs
EMIMAc	1-Ethyl-3-methylimidazolium acetate
DBNHOAc	1,5-diazabicyclo[4.3.0] non-5-enium acetate
